# Supplementary material for: Hydrodynamic pressure law of ground-rested circular RC tank under bi-directional horizontal seismic action
Source: PLoS One. 2022 Mar 22;17(3):e0265528. doi: 10.1371/journal.pone.0265528 (PMC8939809; doi:10.1371/journal.pone.0265528)
Supplement: S1 File — (DOCX) [file pone.0265528.s001.docx]

S1 Table. The maximum hydrodynamic pressure of the tank wall under unidirectional main seismic motion with 1.0 m/s^2^ peak acceleration for A-10% condition (KPa)

| Position | HSDB-EW | JZGYF-NS | PJW-NS | BJ-EW | CC-NS | YL-NS | HX-NS | EL-NS | TJ-NS |
| --- | --- | --- | --- | --- | --- | --- | --- | --- | --- |
| Liquid surface | 0.702 | 1.006 | 1.148 | 0.851 | 1.555 | 1.761 | 2.412 | 0.624 | 0.684 |
| Tank bottom | 0.757 | 1.086 | 1.129 | 0.853 | 1.454 | 1.667 | 2.359 | 0.670 | 0.744 |

S2 Table. The maximum hydrodynamic pressure of the radial direction for tank bottom under unidirectional main seismic motion with 1.0 m/s^2^ peak acceleration for A-10% condition (KPa)

| Position | HSDB-EW | JZGYF-NS | PJW-NS | BJ-EW | CC-NS | YL-NS | HX-NS | EL-NS | TJ-NS |
| --- | --- | --- | --- | --- | --- | --- | --- | --- | --- |
| Radius 1 | 0.757 | 1.060 | 1.129 | 0.853 | 1.454 | 1.479 | 2.359 | 0.670 | 0.645 |
| Position a | 0.380 | 0.722 | 0.674 | 0.647 | 0.521 | 0.779 | 1.707 | 0.294 | 0.414 |
| Position b | 0.333 | 0.636 | 0.620 | 0.761 | 0.979 | 0.960 | 2.103 | 0.381 | 0.434 |
| Position c | 0.365 | 0.514 | 0.991 | 0.767 | 1.182 | 1.161 | 2.010 | 0.420 | 0.504 |
| Position d | 0.391 | 0.338 | 0.901 | 0.755 | 0.728 | 1.030 | 1.649 | 0.800 | 1.092 |
| Center point | 0.037 | 0.001 | 0.019 | 0.070 | 0.045 | 0.046 | 0.050 | 0.091 | 0.088 |
| Position e | 0.322 | 0.236 | 0.021 | 0.814 | 0.820 | 0.932 | 1.237 | 0.746 | 1.233 |
| Position f | 0.252 | 0.419 | 0.700 | 0.850 | 0.912 | 1.499 | 1.520 | 0.518 | 0.379 |
| Position g | 0.321 | 0.599 | 0.700 | 0.805 | 1.194 | 1.306 | 1.593 | 0.437 | 0.378 |
| Position h | 0.344 | 0.750 | 0.651 | 0.372 | 0.971 | 1.001 | 1.253 | 0.397 | 0.465 |
| Radius 2 | 0.618 | 1.086 | 0.976 | 0.805 | 1.072 | 1.667 | 2.068 | 0.644 | 0.744 |

S3 Table. The maximum hydrodynamic pressure of the circumferential direction for tank bottom under unidirectional main seismic motion with 1.0 m/s^2^ peak acceleration for A-10% condition (KPa)

| Angle ($\theta^{\circ}$) | HSDB-EW | JZGYF-NS | PJW-NS | BJ-EW | CC-NS | YL-NS | HX-NS | EL-NS | TJ-NS |
| --- | --- | --- | --- | --- | --- | --- | --- | --- | --- |
| 0°(360°) | 0.757 | 1.060 | 1.129 | 0.853 | 1.454 | 1.479 | 2.359 | 0.670 | 0.645 |
| 22.5° | 0.721 | 1.009 | 1.075 | 0.813 | 1.385 | 1.411 | 2.244 | 0.638 | 0.614 |
| 45° | 0.581 | 0.817 | 0.869 | 0.658 | 1.121 | 1.148 | 1.808 | 0.517 | 0.497 |
| 67.5° | 0.271 | 0.390 | 0.411 | 0.314 | 0.535 | 0.553 | 0.842 | 0.247 | 0.237 |
| 90° | 0.001 | 0.001 | 0.001 | 0.001 | 0.001 | 0.002 | 0.001 | 0.001 | 0.001 |
| 112.5° | 0.229 | 0.402 | 0.362 | 0.298 | 0.398 | 0.630 | 0.771 | 0.239 | 0.279 |
| 135° | 0.478 | 0.839 | 0.755 | 0.623 | 0.829 | 1.298 | 1.604 | 0.498 | 0.577 |
| 157.5° | 0.589 | 1.035 | 0.931 | 0.768 | 1.022 | 1.592 | 1.973 | 0.614 | 0.710 |
| 180° | 0.618 | 1.086 | 0.976 | 0.805 | 1.072 | 1.667 | 2.068 | 0.644 | 0.744 |
| 202.5° | 0.589 | 1.034 | 0.930 | 0.767 | 1.022 | 1.591 | 1.971 | 0.614 | 0.709 |
| 225° | 0.477 | 0.838 | 0.754 | 0.621 | 0.828 | 1.296 | 1.601 | 0.497 | 0.576 |
| 247.5° | 0.298 | 0.523 | 0.472 | 0.388 | 0.517 | 0.817 | 1.004 | 0.311 | 0.362 |
| 270° | 0.077 | 0.135 | 0.122 | 0.100 | 0.134 | 0.213 | 0.255 | 0.080 | 0.094 |
| 292.5° | 0.184 | 0.265 | 0.279 | 0.214 | 0.364 | 0.377 | 0.563 | 0.168 | 0.161 |
| 315° | 0.516 | 0.728 | 0.773 | 0.586 | 0.998 | 1.024 | 1.605 | 0.460 | 0.443 |
| 337.5° | 0.686 | 0.961 | 1.023 | 0.774 | 1.319 | 1.345 | 2.135 | 0.608 | 0.585 |

S4 Table. The maximum hydrodynamic pressure of the tank wall under unidirectional main seismic motion with 1.0 m/s^2^ peak acceleration for A-30% condition (KPa)

| Position | HSDB-EW | JZGYF-NS | PJW-NS | BJ-EW | CC-NS | YL-NS | HX-NS | EL-NS | TJ-NS |
| --- | --- | --- | --- | --- | --- | --- | --- | --- | --- |
| Liquid surface | 0.733 | 1.295 | 1.782 | 0.853 | 3.028 | 7.704 | 3.626 | 1.635 | 1.030 |
| Position 1 | 1.319 | 1.851 | 2.019 | 1.269 | 2.982 | 7.135 | 3.555 | 1.596 | 1.256 |
| Position 2 | 1.766 | 2.272 | 2.183 | 1.610 | 2.853 | 6.672 | 3.502 | 1.824 | 1.599 |
| Tank bottom | 1.892 | 2.391 | 2.226 | 1.710 | 2.825 | 6.534 | 3.488 | 1.986 | 1.754 |

S5 Table. The maximum hydrodynamic pressure of the radial direction for tank bottom under unidirectional main seismic motion with 1.0 m/s^2^ peak acceleration for A-30% condition (KPa)

| Position | HSDB-EW | JZGYF-NS | PJW-NS | BJ-EW | CC-NS | YL-NS | HX-NS | EL-NS | TJ-NS |
| --- | --- | --- | --- | --- | --- | --- | --- | --- | --- |
| Radius 1 | 1.785 | 2.391 | 2.026 | 1.681 | 2.825 | 6.534 | 2.801 | 1.986 | 1.567 |
| Position a | 0.549 | 1.029 | 1.154 | 0.650 | 2.178 | 5.219 | 2.322 | 0.918 | 0.371 |
| Position b | 0.434 | 0.892 | 0.987 | 0.427 | 2.048 | 4.183 | 2.582 | 0.991 | 0.397 |
| Position c | 0.333 | 0.687 | 0.749 | 0.649 | 2.142 | 2.786 | 2.402 | 1.136 | 0.489 |
| Position d | 0.242 | 0.367 | 0.589 | 0.507 | 1.889 | 2.009 | 1.460 | 0.746 | 0.972 |
| Center point | 0.000 | 0.000 | 0.000 | 0.000 | 0.001 | 0.001 | 0.000 | 0.000 | 0.001 |
| Position e | 0.169 | 0.340 | 0.608 | 0.418 | 1.797 | 1.667 | 1.470 | 0.848 | 0.783 |
| Position f | 0.303 | 0.582 | 0.845 | 0.819 | 2.714 | 2.912 | 1.485 | 1.208 | 0.575 |
| Position g | 0.308 | 0.878 | 0.994 | 0.780 | 2.172 | 4.219 | 2.178 | 0.827 | 0.305 |
| Position h | 0.447 | 1.170 | 1.136 | 0.648 | 1.474 | 4.734 | 2.393 | 0.956 | 0.335 |
| Radius 2 | 1.892 | 1.700 | 2.226 | 1.710 | 2.384 | 4.855 | 3.488 | 1.928 | 1.754 |

S6 Table. The maximum hydrodynamic pressure of the circumferential direction for tank bottom under unidirectional main seismic motion with 1.0 m/s^2^ peak acceleration for A-30% condition (KPa)

| Angle ($\theta^{\circ}$) | HSDB-EW | JZGYF-NS | PJW-NS | BJ-EW | CC-NS | YL-NS | HX-NS | EL-NS | TJ-NS |
| --- | --- | --- | --- | --- | --- | --- | --- | --- | --- |
| 0°(360°) | 1.785 | 2.391 | 2.026 | 1.681 | 2.825 | 6.534 | 2.801 | 1.986 | 1.567 |
| 22.5° | 1.700 | 2.277 | 1.930 | 1.601 | 2.691 | 6.224 | 2.666 | 1.892 | 1.493 |
| 45° | 1.377 | 1.841 | 1.563 | 1.295 | 2.177 | 5.037 | 2.152 | 1.532 | 1.209 |
| 67.5° | 0.657 | 0.873 | 0.745 | 0.615 | 1.034 | 2.400 | 1.013 | 0.731 | 0.578 |
| 90° | 0.012 | 0.012 | 0.011 | 0.011 | 0.012 | 0.008 | 0.009 | 0.011 | 0.034 |
| 112.5° | 0.700 | 0.625 | 0.822 | 0.632 | 0.857 | 1.782 | 1.285 | 0.714 | 0.649 |
| 135° | 1.462 | 1.312 | 1.720 | 1.321 | 1.831 | 3.747 | 2.693 | 1.490 | 1.356 |
| 157.5° | 1.804 | 1.620 | 2.122 | 1.630 | 2.270 | 4.627 | 3.325 | 1.838 | 1.672 |
| 180° | 1.892 | 1.700 | 2.226 | 1.710 | 2.384 | 4.855 | 3.488 | 1.928 | 1.754 |
| 202.5° | 1.802 | 1.619 | 2.121 | 1.629 | 2.268 | 4.624 | 3.322 | 1.836 | 1.671 |
| 225° | 1.459 | 1.309 | 1.716 | 1.319 | 1.827 | 3.740 | 2.688 | 1.488 | 1.353 |
| 247.5° | 0.911 | 0.815 | 1.071 | 0.824 | 1.126 | 2.328 | 1.676 | 0.929 | 0.845 |
| 270° | 0.235 | 0.210 | 0.275 | 0.212 | 0.268 | 0.585 | 0.427 | 0.240 | 0.218 |
| 292.5° | 0.447 | 0.591 | 0.507 | 0.417 | 0.702 | 1.633 | 0.682 | 0.497 | 0.393 |
| 315° | 1.226 | 1.638 | 1.392 | 1.152 | 1.937 | 4.485 | 1.913 | 1.364 | 1.077 |
| 337.5° | 1.619 | 2.167 | 1.838 | 1.524 | 2.562 | 5.926 | 2.537 | 1.801 | 1.422 |

S7 Table. The maximum hydrodynamic pressure of the tank wall under unidirectional main seismic motion with 1.0 m/s^2^ peak acceleration for A-70% condition (KPa)

| Position | HSDB-EW | JZGYF-NS | PJW-NS | BJ-EW | CC-NS | YL-NS | HX-NS | EL-NS | TJ-NS |
| --- | --- | --- | --- | --- | --- | --- | --- | --- | --- |
| Liquid surface | 0.956 | 1.251 | 3.210 | 2.971 | 6.555 | 8.268 | 5.186 | 1.958 | 1.403 |
| Position 1 | 1.753 | 1.628 | 3.317 | 3.159 | 6.009 | 8.158 | 5.134 | 1.944 | 1.499 |
| Position 2 | 2.702 | 2.056 | 3.389 | 3.249 | 5.552 | 7.971 | 5.653 | 3.034 | 1.982 |
| Position 3 | 3.363 | 2.705 | 3.423 | 3.319 | 5.389 | 7.768 | 6.106 | 3.758 | 2.609 |
| Position 4 | 3.842 | 3.168 | 3.625 | 3.649 | 5.316 | 7.583 | 6.432 | 4.266 | 3.090 |
| Position 5 | 4.167 | 3.474 | 3.960 | 3.889 | 5.281 | 7.386 | 6.661 | 4.592 | 3.456 |
| Position 6 | 4.355 | 3.647 | 4.196 | 4.042 | 5.268 | 7.266 | 6.804 | 4.768 | 3.705 |
| Tank bottom | 4.417 | 3.701 | 4.280 | 4.099 | 5.266 | 7.130 | 6.856 | 4.819 | 3.804 |

S8 Table. The maximum hydrodynamic pressure of the radial direction for tank bottom under unidirectional main seismic motion with 1.0 m/s^2^ peak acceleration for A-70% condition (KPa)

| Position | HSDB-EW | JZGYF-NS | PJW-NS | BJ-EW | CC-NS | YL-NS | HX-NS | EL-NS | TJ-NS |
| --- | --- | --- | --- | --- | --- | --- | --- | --- | --- |
| Radius 1 | 3.647 | 3.701 | 4.280 | 3.812 | 5.266 | 7.130 | 6.856 | 4.570 | 3.555 |
| Position a | 1.645 | 1.559 | 2.712 | 2.363 | 4.181 | 6.679 | 5.078 | 2.005 | 1.203 |
| Position b | 0.848 | 1.055 | 2.184 | 2.062 | 3.240 | 5.449 | 4.409 | 1.107 | 0.805 |
| Position c | 0.477 | 0.602 | 1.544 | 1.471 | 2.287 | 3.132 | 3.448 | 0.838 | 0.645 |
| Position d | 0.201 | 0.244 | 0.685 | 0.628 | 1.064 | 1.293 | 1.624 | 0.378 | 0.588 |
| Center point | 0.173 | 0.173 | 0.173 | 0.173 | 0.173 | 0.173 | 0.308 | 0.173 | 0.580 |
| Position e | 0.216 | 0.275 | 0.579 | 0.450 | 1.626 | 2.041 | 1.203 | 0.343 | 0.593 |
| Position f | 0.472 | 0.682 | 1.362 | 1.202 | 3.215 | 4.202 | 2.581 | 0.698 | 0.662 |
| Position g | 1.033 | 1.112 | 2.206 | 2.004 | 3.762 | 5.462 | 3.889 | 1.115 | 0.883 |
| Position h | 1.997 | 1.608 | 2.983 | 2.609 | 3.903 | 5.909 | 4.935 | 2.147 | 1.635 |
| Radius 2 | 4.417 | 2.981 | 4.028 | 4.099 | 4.699 | 6.845 | 5.560 | 4.819 | 3.804 |

S9 Table. The maximum hydrodynamic pressure of the circumferential direction for tank bottom under unidirectional main seismic motion with 1.0 m/s^2^ peak acceleration for A-70% condition (KPa)

| Angle ($\theta^{\circ}$) | HSDB-EW | JZGYF-NS | PJW-NS | BJ-EW | CC-NS | YL-NS | HX-NS | EL-NS | TJ-NS |
| --- | --- | --- | --- | --- | --- | --- | --- | --- | --- |
| 0°(360°) | 3.647 | 3.701 | 4.280 | 3.812 | 5.266 | 7.130 | 6.856 | 4.570 | 3.555 |
| 22.5° | 3.474 | 3.525 | 4.076 | 3.631 | 5.016 | 6.791 | 6.529 | 4.353 | 3.386 |
| 45° | 2.813 | 2.854 | 3.301 | 2.941 | 4.061 | 5.496 | 5.280 | 3.524 | 2.741 |
| 67.5° | 1.341 | 1.361 | 1.574 | 1.403 | 1.936 | 2.615 | 2.505 | 1.680 | 1.646 |
| 90° | 0.608 | 0.607 | 0.606 | 0.606 | 0.607 | 0.606 | 0.607 | 0.606 | 1.677 |
| 112.5° | 1.634 | 1.100 | 1.490 | 1.517 | 1.727 | 2.522 | 2.053 | 1.783 | 1.709 |
| 135° | 3.413 | 2.302 | 3.112 | 3.168 | 3.626 | 5.284 | 4.295 | 3.724 | 2.939 |
| 157.5° | 4.211 | 2.842 | 3.840 | 3.907 | 4.478 | 6.524 | 5.300 | 4.594 | 3.626 |
| 180° | 4.417 | 2.981 | 4.028 | 4.099 | 4.699 | 6.845 | 5.560 | 4.819 | 3.804 |
| 202.5° | 4.207 | 2.839 | 3.837 | 3.904 | 4.475 | 6.519 | 5.296 | 4.590 | 3.623 |
| 225° | 3.407 | 2.298 | 3.106 | 3.162 | 3.619 | 5.274 | 4.287 | 3.717 | 2.933 |
| 247.5° | 2.127 | 1.433 | 1.940 | 1.975 | 1.108 | 3.287 | 2.675 | 2.322 | 1.832 |
| 270° | 0.777 | 0.692 | 0.570 | 0.593 | 0.631 | 0.841 | 0.687 | 0.599 | 1.688 |
| 292.5° | 0.913 | 0.926 | 1.071 | 0.955 | 1.318 | 1.777 | 1.699 | 1.143 | 1.656 |
| 315° | 2.504 | 2.541 | 2.939 | 2.619 | 3.616 | 4.892 | 4.698 | 3.138 | 2.440 |
| 337.5° | 3.308 | 3.357 | 3.882 | 3.458 | 4.776 | 6.466 | 6.216 | 4.145 | 3.224 |

S10 Table. The maximum hydrodynamic pressure of the tank wall under unidirectional main seismic motion with 1.0 m/s^2^ peak acceleration for B-70% condition (KPa)

| Position | HSDB-EW | JZGYF-NS | PJW-NS | BJ-EW | CC-NS | YL-NS | HX-NS | EL-NS | TJ-NS |
| --- | --- | --- | --- | --- | --- | --- | --- | --- | --- |
| Liquid surface | 1.154 | 1.022 | 2.844 | 1.825 | 1.763 | 6.251 | 6.298 | 2.077 | 1.992 |
| Position 1 | 1.863 | 1.285 | 3.081 | 2.246 | 2.075 | 5.938 | 5.979 | 2.308 | 1.478 |
| Position 2 | 2.806 | 2.219 | 3.342 | 2.995 | 2.822 | 5.631 | 5.992 | 2.817 | 1.583 |
| Position 3 | 3.459 | 2.893 | 3.517 | 3.707 | 3.387 | 5.110 | 6.029 | 3.363 | 2.246 |
| Position 4 | 3.931 | 3.374 | 3.800 | 4.225 | 3.811 | 4.765 | 6.032 | 3.907 | 2.756 |
| Position 5 | 4.249 | 3.693 | 4.166 | 4.587 | 4.113 | 4.548 | 6.030 | 4.292 | 3.157 |
| Position 6 | 4.433 | 3.873 | 4.390 | 4.809 | 4.301 | 4.429 | 6.030 | 4.531 | 3.434 |
| Tank bottom | 4.493 | 3.930 | 4.468 | 4.888 | 4.368 | 4.206 | 6.033 | 4.616 | 3.541 |

S11 Table. The maximum hydrodynamic pressure of the radial direction for tank bottom under unidirectional main seismic motion with 1.0 m/s^2^ peak acceleration for B-70% condition (KPa)

| Position | HSDB-EW | JZGYF-NS | PJW-NS | BJ-EW | CC-NS | YL-NS | HX-NS | EL-NS | TJ-NS |
| --- | --- | --- | --- | --- | --- | --- | --- | --- | --- |
| Radius 1 | 3.673 | 3.930 | 4.468 | 4.888 | 4.368 | 4.206 | 6.033 | 4.616 | 3.541 |
| Position a | 2.315 | 2.393 | 2.848 | 3.093 | 2.837 | 3.615 | 5.027 | 2.815 | 1.865 |
| Position b | 1.382 | 1.370 | 2.022 | 1.886 | 1.808 | 2.648 | 3.979 | 1.766 | 0.977 |
| Position c | 0.757 | 0.720 | 1.283 | 1.059 | 1.076 | 1.922 | 2.754 | 1.085 | 0.848 |
| Position d | 0.303 | 0.278 | 0.565 | 0.431 | 0.459 | 0.981 | 1.268 | 0.464 | 0.787 |
| Center point | 0.239 | 0.239 | 0.239 | 0.239 | 0.239 | 0.239 | 0.239 | 0.239 | 0.777 |
| Position e | 0.364 | 0.300 | 0.510 | 0.385 | 0.395 | 0.973 | 1.075 | 0.448 | 0.801 |
| Position f | 0.917 | 0.624 | 1.133 | 0.904 | 0.777 | 1.900 | 2.418 | 0.995 | 0.994 |
| Position g | 1.685 | 1.062 | 1.683 | 1.483 | 1.015 | 2.731 | 3.640 | 1.851 | 1.622 |
| Position h | 2.829 | 1.709 | 2.564 | 2.170 | 1.863 | 3.285 | 4.776 | 3.117 | 2.564 |
| Radius 2 | 4.493 | 2.629 | 4.040 | 3.027 | 3.454 | 3.838 | 5.909 | 4.914 | 4.127 |

S12 Table. The maximum hydrodynamic pressure of the circumferential direction for tank bottom under unidirectional main seismic motion with 1.0 m/s^2^ peak acceleration for B-70% condition (KPa)

| Angle ($\theta^{\circ}$) | HSDB-EW | JZGYF-NS | PJW-NS | BJ-EW | CC-NS | YL-NS | HX-NS | EL-NS | TJ-NS |
| --- | --- | --- | --- | --- | --- | --- | --- | --- | --- |
| 0°(360°) | 3.673 | 3.930 | 4.468 | 4.888 | 4.368 | 4.206 | 6.033 | 4.616 | 3.541 |
| 22.5° | 3.500 | 3.745 | 4.258 | 4.658 | 4.162 | 4.007 | 5.748 | 4.399 | 3.374 |
| 45° | 2.836 | 3.034 | 3.450 | 3.774 | 3.372 | 3.245 | 4.653 | 3.563 | 2.734 |
| 67.5° | 1.773 | 1.896 | 2.157 | 2.360 | 2.108 | 2.027 | 2.903 | 2.227 | 1.709 |
| 90° | 0.480 | 0.480 | 0.480 | 0.480 | 0.480 | 0.480 | 0.480 | 0.480 | 1.496 |
| 112.5° | 1.657 | 0.968 | 1.490 | 1.117 | 1.276 | 1.412 | 2.164 | 1.812 | 1.527 |
| 135° | 3.082 | 1.802 | 2.771 | 2.076 | 2.371 | 2.631 | 4.043 | 3.371 | 2.830 |
| 157.5° | 4.074 | 2.383 | 3.663 | 2.745 | 3.132 | 3.479 | 5.354 | 4.456 | 3.741 |
| 180° | 4.493 | 2.629 | 4.040 | 3.027 | 3.454 | 3.838 | 5.909 | 4.914 | 4.127 |
| 202.5° | 4.282 | 2.505 | 3.850 | 2.885 | 3.292 | 3.657 | 5.629 | 4.683 | 3.933 |
| 225° | 3.469 | 2.029 | 3.119 | 2.337 | 2.668 | 2.962 | 4.554 | 3.794 | 3.186 |
| 247.5° | 2.169 | 1.267 | 1.950 | 1.461 | 1.669 | 1.849 | 2.838 | 2.372 | 1.992 |
| 270° | 0.480 | 0.480 | 0.480 | 0.480 | 0.480 | 0.480 | 0.480 | 0.480 | 1.496 |
| 292.5° | 1.355 | 1.449 | 1.648 | 1.803 | 1.610 | 1.547 | 2.215 | 1.702 | 1.465 |
| 315° | 2.519 | 2.695 | 3.065 | 3.353 | 2.995 | 2.882 | 4.132 | 3.166 | 2.428 |
| 337.5° | 3.330 | 3.563 | 4.051 | 4.239 | 3.960 | 3.812 | 5.468 | 4.185 | 3.210 |

S13 Table. The maximum hydrodynamic pressure of the tank wall under unidirectional main seismic motion with 1.0 m/s^2^ peak acceleration for C-70% condition (KPa)

| Position | HSDB-EW | JZGYF-NS | PJW-NS | BJ-EW | CC-NS | YL-NS | HX-NS | EL-NS | TJ-NS |
| --- | --- | --- | --- | --- | --- | --- | --- | --- | --- |
| Liquid surface | 1.394 | 2.249 | 1.520 | 1.062 | 1.684 | 6.211 | 2.946 | 0.925 | 1.282 |
| Position 1 | 2.051 | 2.610 | 1.813 | 1.395 | 1.669 | 6.002 | 3.202 | 1.462 | 1.074 |
| Position 2 | 2.688 | 3.076 | 2.453 | 2.204 | 2.185 | 6.156 | 3.694 | 2.409 | 1.595 |
| Position 3 | 3.113 | 3.452 | 3.100 | 2.856 | 2.891 | 6.285 | 4.285 | 3.091 | 2.171 |
| Position 4 | 3.544 | 3.721 | 3.601 | 3.351 | 3.451 | 6.371 | 4.873 | 3.610 | 2.630 |
| Position 5 | 3.856 | 3.900 | 3.968 | 3.712 | 3.863 | 6.426 | 5.269 | 3.993 | 3.007 |
| Position 6 | 4.039 | 4.000 | 4.201 | 3.944 | 4.126 | 6.458 | 5.216 | 4.240 | 3.279 |
| Tank bottom | 4.100 | 4.031 | 4.284 | 4.028 | 4.722 | 6.670 | 4.907 | 4.331 | 3.387 |

S14 Table. The maximum hydrodynamic pressure of the radial direction for tank bottom under unidirectional main seismic motion with 1.0 m/s^2^ peak acceleration for C-70% condition (KPa)

| Position | HSDB-EW | JZGYF-NS | PJW-NS | BJ-EW | CC-NS | YL-NS | HX-NS | EL-NS | TJ-NS |
| --- | --- | --- | --- | --- | --- | --- | --- | --- | --- |
| Radius 1 | 3.675 | 4.031 | 4.284 | 4.028 | 4.722 | 6.670 | 4.907 | 4.331 | 3.387 |
| Position a | 1.265 | 2.012 | 1.415 | 0.826 | 1.571 | 5.828 | 2.015 | 0.907 | 0.737 |
| Position b | 0.801 | 1.576 | 1.164 | 0.630 | 2.046 | 4.654 | 1.684 | 0.648 | 0.539 |
| Position c | 0.588 | 1.244 | 1.268 | 0.753 | 1.373 | 3.683 | 2.384 | 0.519 | 0.504 |
| Position d | 0.414 | 0.721 | 0.813 | 0.684 | 1.783 | 3.103 | 1.902 | 0.614 | 0.511 |
| Center point | 0.134 | 0.134 | 0.134 | 0.134 | 0.134 | 0.134 | 0.134 | 0.134 | 0.497 |
| Position e | 0.387 | 0.759 | 0.689 | 0.615 | 1.641 | 2.902 | 2.872 | 0.615 | 0.498 |
| Position f | 0.590 | 1.219 | 0.747 | 0.781 | 1.176 | 4.709 | 3.216 | 0.452 | 0.506 |
| Position g | 0.821 | 1.560 | 1.004 | 0.881 | 1.828 | 4.687 | 3.222 | 0.549 | 0.546 |
| Position h | 1.441 | 1.936 | 1.174 | 1.028 | 1.845 | 5.251 | 2.561 | 0.878 | 0.771 |
| Radius 2 | 4.100 | 3.622 | 3.518 | 3.213 | 3.174 | 5.949 | 4.888 | 4.612 | 3.667 |

S15 Table. The maximum hydrodynamic pressure of the circumferential direction for tank bottom under unidirectional main seismic motion with 1.0 m/s^2^ peak acceleration for C-70% condition (KPa)

| Angle ($\theta^{\circ}$) | HSDB-EW | JZGYF-NS | PJW-NS | BJ-EW | CC-NS | YL-NS | HX-NS | EL-NS | TJ-NS |
| --- | --- | --- | --- | --- | --- | --- | --- | --- | --- |
| 0°(360°) | 3.675 | 4.031 | 4.284 | 4.028 | 4.722 | 6.670 | 4.907 | 4.331 | 3.387 |
| 22.5° | 3.502 | 3.841 | 4.082 | 3.839 | 4.022 | 6.165 | 3.427 | 4.128 | 3.231 |
| 45° | 2.836 | 3.110 | 3.307 | 3.110 | 3.259 | 4.995 | 2.777 | 3.345 | 2.613 |
| 67.5° | 1.772 | 1.941 | 2.068 | 1.945 | 2.038 | 3.123 | 1.736 | 2.092 | 1.636 |
| 90° | 0.801 | 0.801 | 0.801 | 0.801 | 0.801 | 0.801 | 0.801 | 0.801 | 1.660 |
| 112.5° | 1.512 | 1.334 | 1.297 | 1.185 | 1.173 | 2.187 | 1.792 | 1.702 | 1.690 |
| 135° | 2.812 | 2.483 | 2.413 | 2.203 | 2.179 | 4.076 | 3.350 | 3.164 | 2.514 |
| 157.5° | 3.717 | 3.283 | 3.190 | 2.913 | 2.879 | 5.392 | 4.431 | 4.181 | 3.324 |
| 180° | 4.100 | 3.622 | 3.518 | 3.213 | 3.174 | 5.949 | 4.888 | 4.612 | 3.667 |
| 202.5° | 3.907 | 3.451 | 3.353 | 3.061 | 3.025 | 5.668 | 4.658 | 4.395 | 3.494 |
| 225° | 3.165 | 2.795 | 2.716 | 2.480 | 2.452 | 4.589 | 3.772 | 3.561 | 2.830 |
| 247.5° | 1.979 | 1.747 | 1.698 | 1.551 | 1.534 | 2.865 | 2.352 | 2.227 | 1.770 |
| 270° | 0.801 | 0.801 | 0.801 | 0.801 | 0.801 | 0.801 | 0.801 | 0.801 | 1.660 |
| 292.5° | 1.354 | 1.481 | 1.580 | 1.486 | 1.557 | 2.386 | 1.326 | 1.599 | 1.630 |
| 315° | 2.519 | 2.762 | 2.938 | 2.764 | 2.896 | 4.438 | 2.468 | 2.972 | 2.322 |
| 337.5° | 3.331 | 3.654 | 3.884 | 3.652 | 3.826 | 5.866 | 3.261 | 3.927 | 3.073 |
